# Building a maternal and child cohort amidst Lebanon’s socioeconomic collapse: preliminary results and navigating research challenges

**DOI:** 10.1186/s12963-024-00325-1

**Published:** 2024-03-26

**Authors:** Rima Kaddoura, Martine Elbejjani, Hani Tamim, Ziyad R. Mahfoud, Pascale Salameh, Fadi Mirza, Lama Charafeddine

**Affiliations:** 1 American University of Beirut (AUB), Beirut, Lebanon; 2 Alfaisal University, Riyadh, Kingdom of Saudi Arabia; 3 Weill Cornell Medicine-Qatar, Al Rayyan, Qatar; 4 Faculty of Pharmacy, Lebanese University, Hadat, Lebanon; 5 School of Medicine, Lebanese American University, Byblos, Lebanon; 6 Institut National de Santé Publique d’Épidémiologie Clinique et de Toxicologie-Liban (INSPECT-LB), Beirut, Lebanon; 7 Department of Primary Care and Population Health, University of Nicosia Medical School, 2417 Nicosia, Cyprus; 8 Latifa Hospital & Private Practice, Dubai, United Arab Emirates

**Keywords:** Maternal fetal cohort, Preterm birth, Research challenges, Socioeconomic factors, Lebanon

## Abstract

The impact of conflict and crisis on maternal and child health underscores the need for reliable research in vulnerable populations. Lebanon, amidst ongoing economic collapse, political instability, and healthcare system strain, offers a case study for exploring these impacts, particularly on preterm babies and their development. This study aims to assess the feasibility of establishing a prospective cohort of mothers and their full-term and preterm babies in Lebanon, examining the association between social determinants, preterm birth, and developmental outcomes amidst the nation's multifaceted crises. The planned cohort involves 50 full-term and 50 preterm mother-baby pairs recruited at birth and followed up to 9–12 months post-birth. Data collection spans social determinants, perceived stress, social support, quality of life, and developmental assessments. Challenges in recruitment, follow-up, and data collection in the context of Lebanon's socio-political and economic turmoil are evaluated, alongside ethical considerations for research in vulnerable populations. Preliminary findings highlight substantial recruitment and follow-up challenges, notably due to population mobility, economic instability, and healthcare access issues. Despite these obstacles, 113 mother-baby pairs have been recruited. Early analysis reveals significant stress and reduced quality of life among mothers, particularly those with preterm infants, against a backdrop of declining birth rates and healthcare worker exodus. Conducting research in crisis settings like Lebanon presents unique methodological and ethical challenges but remains crucial for understanding and improving health outcomes in vulnerable populations. The study underscores the importance of adaptable research designs and ethical diligence in crisis research, highlighting the need for interventions tailored to these contexts. Establishing a mother and child cohort in Lebanon's crisis-ridden setting is faced with many challenges but is essential for guiding future interventions. Research in such contexts is needed to address health disparities and supporting vulnerable populations, emphasizing the need for dedicated funding and innovative research approaches in times of crisis.

## Background

Maternal and child health is an essential area of population health which is disproportionately affected by conflict and crisis situations. These situations heighten existing vulnerabilities and pose unique challenges to health research [12]. Methodological issues arise which could impact the reliability of data being collected due to population displacement and immigration and potential destructions of health systems [34]. Longitudinal studies become particularly impacted with an increased risk of loss to follow-up and increasing difficulty of maintaining the data collection system in place [5]. The implementation of health research methods and ethical requirements ought to be specifically considered given the increased vulnerability of the population in crisis settings [19]. Despite these obstacles, such research is essential for informing interventions that can significantly improve health outcomes in some of the most vulnerable populations. For the past few years, Lebanon has been an unfortunate perfect case study of a country being faced with multiple simultaneous crises. Economically, the financial crisis which started in late 2019 is still ongoing and is manifesting in the depreciation of the local currency at more than 90% its value, in an increasingly soaring inflation at more than 200%, and in significant restrictions on banking transactions and funds use [6]. From a political perspective, since 2019, Lebanon has been gripped by a national revolution, political unrest, presidential vacuum, and widespread corruption across multiple public sectors [22]. From a public perspective, lack of essential services from electricity to fuel, to medications, has severely impacted the lives of Lebanon’s residents for the past several years [15].

Lebanon’s infrastructure, particularly healthcare infrastructure, has also been further strained by the impact of the COVID-19 pandemic, the financial challenges, and the catastrophic August 2020 Beirut port explosion. The healthcare crisis is multifaceted and has been characterized by staffing shortage due to the exodus of healthcare workers [29] closure of medical centers [7], shortage of medications, and shortage of instruments and lab kits [28] to name a few.

In this context, exploring the impact of crises on vulnerable groups becomes imperative. Preterm babies and their parents are already at a disadvantage with a need for medical, social, and emotional support especially during the critical 1000 days for them to reach their developmental potential. Several social and structural risk factors have been identified for preterm birth. Some of the main determinants of preterm birth are social disadvantage and low socioeconomic conditions [2, 21, 38], lower mothers’ educational status [14], and higher maternal anxiety and stress in pregnancy [13]. Structural factors such as income and mother’s educational level are not only directly influential, but also impact preterm birth through other intermediary factors [14]. Structural barriers and limited or inadequate access to health services are also risk factors for preterm birth [11]. Other studies have also shown that lower income is among the structural factors which affect preterm birth, and perceived stress, perceived social support and financial support are also associated with preterm birth [9, 14, 20, 24, 40]. In addition, studies have revealed that preterm birth is more common in mothers who suffer from anxiety and depression [20, 31, 39, 40].In Lebanon, the deterioration of the healthcare and economic systems is expected to have had large negative consequences on this vulnerable group. Indeed, economic disadvantage is not only a structural risk factor of preterm birth, but also of other important developmental outcomes, including cognitive functioning, and social, behavioral, and emotional development [27, 36, 37].

Cohort studies are distinctive in supporting our understanding of child development, particularly in contexts like Lebanon, allowing for observation and follow-up for an extended period, capturing dynamic changes and long-term outcomes. Lebanon's multifaceted crises, which encompass economic downturns, political gridlock, infrastructural decay, and social unrest, serve as an important case for studying the impact of the social environment and structural determinants of health on vulnerable preterm infants' developmental outcomes. The life course theory as a framework can provide a comprehensive insight to examine how our cohort is affected by the interplay of life events, social conditions, and historical changes and how these can influence individual development over time [16, 17]. Thus, exploring the feasibility of implementing a cohort study in a crisis setting in Lebanon sets the stage for further similar health research.

## Objectives

We originally aimed at building a cohort of 50 full term babies and their mothers and 50 pre-term babies and their mothers, recruited at birth, and followed up for up to 9–12 months post birth. The objectives of this cohort were to examine the association between social determinants and preterm birth, and to examine the association between preterm birth and developmental outcomes of infants. In parallel with the launch and establishment of the cohort, Lebanon’s rapid socio-political changes and complex financial and healthcare challenges began unfolding.

In this report, the objectives are to examine the feasibility of building a prospective mother and child cohort, and to examine the challenges and barriers of conducting such research in a crisis ridden and low resource setting.

### Population and sample size

The study population of the originally planned cohort consisted of babies born before 37 weeks of gestation at the American University of Beirut Medical Center (AUBMC) and their mothers, and a comparator group of babies born at 37 weeks of gestation and above also born at AUBMC. Based on a review of the literature of previous studies comparing Bayley scales between children born at term and children born preterm, we estimated the sample size needed to be able to reject the null hypothesis that preterm children and full-term children will have similar Bayley scores at 9–12 months post birth.

We thus planned a study of a continuous response variable from independent control (full term children) and experimental subjects (preterm children) with 1 control per experimental subject. In a previous study the response within each subject group was normally distributed with standard deviation 3.4 [26]. If the true difference in the experimental and control means is 2.1 via t-test, we will need to study 48 experimental subjects and 48 control subjects to be able to reject the null hypothesis that the population means of the experimental and control groups are equal with probability (power) 0.85. Thus, we aimed at recruiting 50 full term babies and 50 preterm babies and their mothers. The Type I error probability associated with this test of this null hypothesis is 0.05. After the inclusion of two additional study centers, and taking into account the participants’ drop out at follow-up visits, the target sample was increased to 80 full term babies and 80 preterm babies and their mothers.

Non-Lebanese and those who are not permanent residents in Lebanon were excluded as these are likely to be the most mobile thus limiting the possibility of follow up and leading to high attrition rates. Whilst babies born to Lebanese moms, but not Lebanese fathers are considered non-Lebanese but do obtain residencies, these were excluded as this population could have particular characteristics (such as limitations relating to father’s employment and insurance limitations) which would differentiate them from the rest of the sample and could be the source of potential confounding factors. Also excluded were infants with major congenital anomalies (Trisomy 13, Trisomy 18, Holoprosencephaly, Anencephaly, Encephalocele) who exhibit severely impaired communication as they would be unable to undergo the needed developmental assessment during follow-up visits. Finally, babies who passed away in the Neonatal Intensive Care Unit (NICU) were excluded.

### Design

The original cohort was a single center prospective cohort. It was expanded to a multi-center cohort later due to lack of recruitment. Recruitment at AUBMC was launched in September 2021, at Keserwan Medical Center (KMC) in June 2022, and at Bahman Hospital in October 2022. The original plan was for one year of data collection done at three time points: At recruitment in the postpartum unit after birth, at 4–6 months post birth, and at 9–12 months post birth.

The project has been approved by the Institutional Review Board (IRB) of all participating centers and informed consent from the mothers is secured upon recruitment prior to inclusion in the study.

## Methods

The cohort aims at collecting information on several aspects including social determinants, perceived stress level, social support, and quality of life. In addition, developmental assessment of all babies is performed at 4–6 months post birth and at 9–12 months post birth. These are presented in further detail below:

The social determinants assessed include maternal age, educational level, city of residence, and household crowding index. Given that participants might not be willing/able to disclose their income, income is measured via both a direct question and via proxy variables including ability to acquire basic needs (food, shelter, clothing, sanitation, education, and healthcare); assets owned; and perception of wealth inequality through the society ranking scale.

Also assessed are the mothers’ perceived stress level, social support, and quality of life at birth, at 4–6 months, and at 9–12 months using the following validated and standardized tools:

- Arabic version of the Cohen perceived stress scale (PSS-10), a validated tool used to measure stress during pregnancy and the postpartum period. The tool generates a 10-item scale which correlates with depression, anxiety, decreased satisfaction, and perception of poor health. It has been translated and validated in Arabic in a Lebanese population sample.
- Arabic version of the Multidimensional Scale of Perceived Social Support (MSPSS), a tool of 12 items that assesses social support in the individual’s community consisting of family, friends, and significant others. It has been translated and validated in Arabic in a Lebanese population sample.
- Arabic version of the WHO Quality of Life Instrument-Bref (WHOQoL‐BREF). The questionnaire measures physical and psychological health, social relationships, and the environment and has been validated in an Arabic population.

Developmental assessment of all babies is performed at two time points: at the 4–6 months and at 9–12 months visit using the Ages & Stages Questionnaire. The Bayley Scales of Infant & Toddler Development is administered at the 9-12months visit. The Ages & Stages Questionnaires is a developmental screening tool for developmental delays. The questionnaire assesses 5 areas of development (Communication, gross motor, fine motor, problem solving, personal-social) with 6 questions/area. Depending on the score generated, the results indicate either that the child’s development is on schedule, and they receive a result of “pass”, either that there are behaviors of concern, and the child should be “provided activities and retested”, either that there is concern and further assessment by a professional is needed and they receive a result of “refer”. It has been tested in the Lebanese population with adequate reliability and validity [8]. The Bayley Scale for Infant and Toddler Development is recognized internationally as a gold standard test to assess development,it is administered in English by a certified trained professional.

Finally, clinical and health related variables are collected through medical records review. These include medical history, and pregnancy and delivery history. Based on a review of the literature, data is also collected on the following potential confounders to be adjusted for in the analysis: gender; neonatal morbidities defined as: sepsis, intraventricular hemorrhage, and respiratory distress syndrome; parity; multiple births; babies undergoing physiotherapy; and babies undergoing speech therapy.

### Feasibility assessment

Whilst the project is not structured as a feasibility study, we have referred to feasibility assessment methods to track and evaluate the challenges faced throughout the study duration as reported by Osmond and Cohn [30]. Our assessment methods focused on the following objectives:

1. Evaluation of recruitment feasibility and how would recruitment challenges impact the sample characteristics. Measures of interest are speed of recruitment, number recruited, and characteristics of the recruited sample and whether these are expected and representative of the planned sample.
2. Evaluation of data collection measures and how would limitations in data collection impact measurements of both the outcome and risk factors. Measures of interest include the ability to collect data on the variables of interest whether these are risk factors or outcomes and if they are aligned with the planned data collection measures.
3. Evaluation of available resources and how would challenges impact the study implementation and progress. Measures of interest include availability of resources (whether financial, logistic, or staff and researchers’ availability) and whether these are impacting the ability to implement and progress with the study as planned (Table 1).

**Table 1:** Feasibility assessment and proposed recommendations

Feasibility assessment	Challenges faced	Addressing the challenges	Recommendations
Evaluation of recruitment feasibility and how would recruitment challenges impact the sample characteristics	Lower numbers recruited than expected	Addition of further centres to support recruitment efforts	Targeted recruitment efforts (community-based recruitment, collaboration with non-governmental organizations,outpatient health centers…)
			Analyze characteristics of those lost to follow up
	Participants not meeting eligibility criteria	Hiring additional research personnel	Build a representative sample which includes all socioeconomic levels
Evaluation of available resources and how would challenges impact the study implementation and progress	Rising transportation costs	Scheduling follow-up visits alongside the paediatrician’s visit to limit the need for transportation to the medical centre	Scheduling study related visits alongside healthcare follow-up visit
	Rising charges for consultation visits	Hiring additional research personnel	Providing transportation fees
Evaluation of data collection measures and how would limitations in data collection impact measurements of both the outcome and risk factors	Limited and inaccurate data on finances	Collect data on additional measures of financial capabilities: assets owned, ability to acquire basic needs, perception of wealth inequality through society ranking scale	Collecting data on proxy measures of financial capabilities

### Statistical analysis

This manuscript presents descriptive statistics and preliminary results and how these relate to the challenges faced. SPSS version 29 was used to perform descriptive analyses which consisted of calculating means for continuous variables and frequencies and percentages for categorical variables.

## Results

As per the feasibility assessment framework, which was presented, a summary of the challenges faced, ways of addressing these challenges, and suggested recommendations are summarized in Table 2 and further discussed below.

**Table 2:** Baseline sample characteristics of 113 Lebanese mother-baby pairs enrolled in the cohort

Characteristics at baseline for categorical variables	n (%)				
*Mother's Educational level*	*Full term (n* = *79)*		*Preterm (n* = *34)*		Total
Secondary	5	6.3%	4	11.8%	9
Technical	2	2.5%	0	0.0%	2
Undergraduate	18	22.8%	7	20.6%	25
Graduate	53	67.1%	23	67.6%	76
Total	78	98.7%*	34	100.0%	112
*Mother's Occupation*					
Unemployed	39	49.3%	17	50%	56
Self-employed	4	5.1%	3	8.80%	7
Employed part time	4	5.1%	1	2.90%	5
Employed full time	32	40.5%	13	38.20%	45
Total	79	100.0%	34	100.0%	113
*Father's Occupation*					
Unemployed	0	0.0%	1	2.9%	1
Self-employed	16	20.2%	11	32.4%	27
Employed part time	4	5.1%	0	0.0%	4
Employed full time	59	74.7%	22	64.7%	81
Total	79	100.0%	34	100.0%	113
*Mother's insurance status*					
Uninsured	2	2.5%	4	11.8%	6
National Social Security Fund (NSSF)	11	13.9%	3	8.8%	14
Health Insurance Plan (HIP)	12	15.2%	4	11.8%	16
Private Insurance	54	68.4%	23	67.6%	77
Total	79	100.0%	34	100.0%	113
*Complications during pregnancy*					
No	58	73.4%	11	32.3%	69
Yes	20	25.3%	23	67.7%	43
Total	78	98.7%*	34	100.0%	112
*Mother's comorbidities*					
No	61	77.2%	24	70.60%	85
Yes	17	21.5%	10	29.40%	27
Total	78	98.7%*	34	100.00%	112
*Baby's gender*					
Male	44	55.7%	19	55.90%	63
Female	34	43.0%	15	44.10%	49
Total	78	98.7%*	34	100.00%	112
*Multiple births*					
Singleton	74	93.6%	18	52.90%	92
Twins or more	4	5.1%	16	47.10%	20
Total	78	98.7%*	34	100.00%	112
*Mode of birth*					
Normal Vaginal Delivery	47	59.5%	8	23.50%	55
Planned C-section	22	27.8%	6	17.60%	28
Urgent C-section	9	11.4%	20	58.90%	29
Total	78	98.7%*	34	100.00%	112
*Growth classification (10th percentile)*					
Appropriate for Gestational Age (AGA)	65	82.3%	25	73.50%	90
Small for Gestational Age (SGA)	1	1.2%	0	0.00%	1
Large for Gestational Age (LGA)	12	15.2%	9	26.50%	21
Total	78	98.7%*	34	100.00%	112
*Feeding practices*					
Breastmilk	43	54.4%	4	11.80%	47
Formula	3	3.8%	6	17.60%	9
Mixed	17	21.5%	20	58.80%	37
Total	63	79.7%**	30	88.2%**	93
*Ability to acquire food*					
No	0	0.0%	1	2.90%	1
Yes	79	100.00%	33	97.10%	112
Total	79	100.00%	34	100.00%	113
*Ability to acquire clothing*					
No	2	2.5%	2	5.90%	4
Yes	77	97.5%	32	94.10%	109
Total	79	100.00%	34	100.00%	113
*Ability to acquire shelter*					
No	0	0.0%	2	5.90%	2
Yes	79	100.00%	32	94.10%	111
Total	79	100.00%	34	100.00%	113
*Ability to acquire sanitation*					
No	0	0.0%	1	2.90%	1
Yes	79	100.00%	33	97.10%	112
Total	79	100.00%	34	100.00%	113
*Ability to acquire education*					
No	0	0.0%	3	8.80%	3
Yes	61	77.2%	17	50.00%	78
Not Applicable	18	22.8%	14	41.20%	32
Total	79	100.00%	34	100.00%	113
*Ability to acquire healthcare*					
No	1	1.3%	2	5.90%	3
Yes	78	98.7%	32	94.10%	110
Total	79	100.00%	34	100.00%	113
*Do you own a car*					
No	2	2.5%	5	14.70%	7
Yes	77	97.5%	29	85.30%	106
Total	79	100.00%	34	100.00%	113
*Do you own a house*					
No	16	20.3%	10	29.40%	26
Yes	63	79.7%	24	70.60%	87
Total	79	100.00%	34	100.00%	113
*Do you own land*					
No	50	63.3%	28	82.40%	78
Yes	29	36.7%	6	17.60%	35
Total	79	100.00%	34	100.00%	113
*PSS10*					
Low	34	43.0%	8	23.50%	42
Moderate	36	45.6%	18	52.90%	54
High	9	11.4%	8	23.50%	17
Total	79	100.00%	34	100.00%	113
*MSPSS*					
Low	0	0.0%	0	0.00%	0
Moderate	11	13.9%	6	17.60%	17
High	68	86.1%	28	82.40%	96
Total	79	100.00%	34	100.00%	113

^*^Missing data for one full-term baby

^**^Missing data for 16 full-term and 4 pre-term babies

### Challenges in recruitment

We were initially expecting to recruit 100 mother-baby pairs in the first year of the study, we were only able to recruit 70 mother-baby pairs by the end of the first year. Although some of the mothers who were approached did not want to partake in the study, a significant number of those who were interested were not eligible to be included in the cohort, thus decreasing the sample pool. Specifically, 21 mothers who were approached could not be recruited since they were not permanent residents in Lebanon although they expressed interest in taking part in the study. In terms of center distribution, 95% were recruited from AUBMC, 4% were recruited from Bahman hospital, and 1% were recruited from KMC, although these percentages are expected to change with the ongoing recruitment. Thus, with the overall majority being recruited from one center only, a comparison of the characteristics and sample distribution between centers was not considered.

### Challenges in follow-up assessment

Our study is a longitudinal cohort with follow up assessments scheduled at two time points after birth, at 4–6 months, and at 9–12 months. While a common limitation of all cohorts is the loss to follow up, we have noted that in our sample several of the women who are not completing the follow up visits are not withdrawing due to lack of interest in partaking. Rather, rising transportation costs and rising charges for consultation visits are impacting the ability to complete follow-up assessments. To date, only 71% of the mothers who have reached the follow-up stage have completed the first follow-up visit. For those who did not complete the follow-up visit, and whom we were able to contact, around 40% stated the rising fuel costs, inability to cover transportation costs, and inability to come to AUBMC for follow up as the main reason for withdrawal.

### Challenges in data collection

One of the main challenges we have faced is in collecting data on finances and economic resources. In the cohort, 40% of the mothers were not able to disclose their income, partly because they are getting paid part of their salaries in USD and could not calculate the equivalent in LBP. It was frequently difficult for both the participants and for the purpose of the study to calculate the monthly income due to multiple reasons:

- The value of the Lebanese Lira was officially pegged at 1 USD = 1,500 L.L for most of the economic crisis despite the devaluation in the actual market.
- The value of the Lebanese Lira fluctuated frequently from day to day and sometimes during the same day.
- There was no official source for the value of the Lebanese Lira to the USD. Values were deducted from blogs, exchange shops, and social media reporting.
- Income varied between different institutions in the country in which there were several options to receive income by an employee: Either fully in USD, or fully in LBP at the market rate, or partially in USD, partially in LBP at market rate and partially in LBP at the bank rate.

With the lack of official tracking systems available to monitor the value of the Lebanese Lira, this has impacted the socioeconomic status evaluation of the mothers in our sample. Issues in data collection due to limited resources and nation-wide barriers has been reported in neighbouring countries including the occupied Palestinian territory [23].

### Addressing the challenges

To mitigate the effects of these issues in recruitment, follow-up, and data collection process, we implemented some changes in terms of study site, follow-up process, and study personnel. The cohort was originally planned in one center only; however, nine months after recruitment was launched at AUBMC, and to support recruitment efforts, particularly for preterm infants, another center, Keserwan Medical Center (KMC), was added as a recruitment center. A year after recruitment launched, a third center, Bahman Hospital was also added, to both support recruitment efforts, and to extend the sampling frame to those of lower socioeconomic levels. The inclusion and exclusion criteria were the same across the different centers. AUBMC serves a diverse population of patients, including the more central and urban residents of the capital Beirut, as well as employees of the institution who benefit from the Health Insurance Plan (HIP) and who have diverse socioeconomic levels, in addition to the outpatient clinic which specializes in offering services for residents with limited financial or healthcare coverage resources. KMC is located to the north of Lebanon and serves residents who are further from the capital. Bahman offers medical care to residents with limited resources for medical care support and financial means.

After the inclusion of the two additional sites, the target sample was increased to 80 full term babies and 80 preterm babies and their mothers. At the time of continuing review, permission was granted from the Institutional Review Board to extend recruitment at all sites considering the challenges being faced particularly regarding the decreasing number of admissions at the hospitals.

In terms of follow-up visits, these are being scheduled alongside the children’s paediatrician’s visit to limit the need for transportation to the medical center for the mothers. In the event of mothers choosing to follow up with paediatricians in other centers that are not part of the sample, they are being granted the opportunity of completing the follow-up questionnaires over the phone thus limiting the rate of missing data. Finally, an additional research assistant was hired to support with recruitment and follow-up efforts across the centers.

### Preliminary analysis

**Fig. 1:**
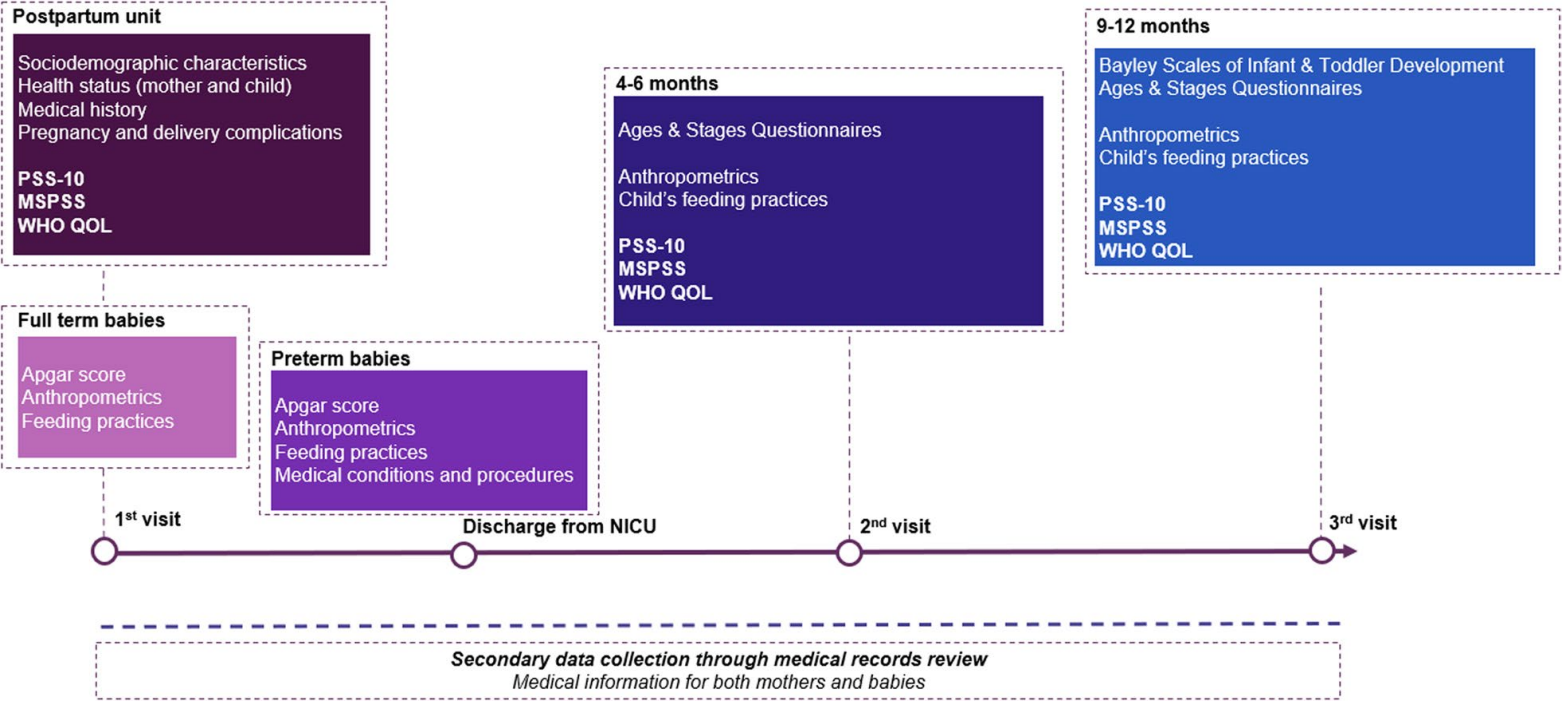
Data collection timeline

The preliminary results reported at this stage consist of basic descriptive statistics and the profile of the study population at baseline. A timeline of the study and the data collection schedule is detailed in Fig. 1.

**Fig. 2:**
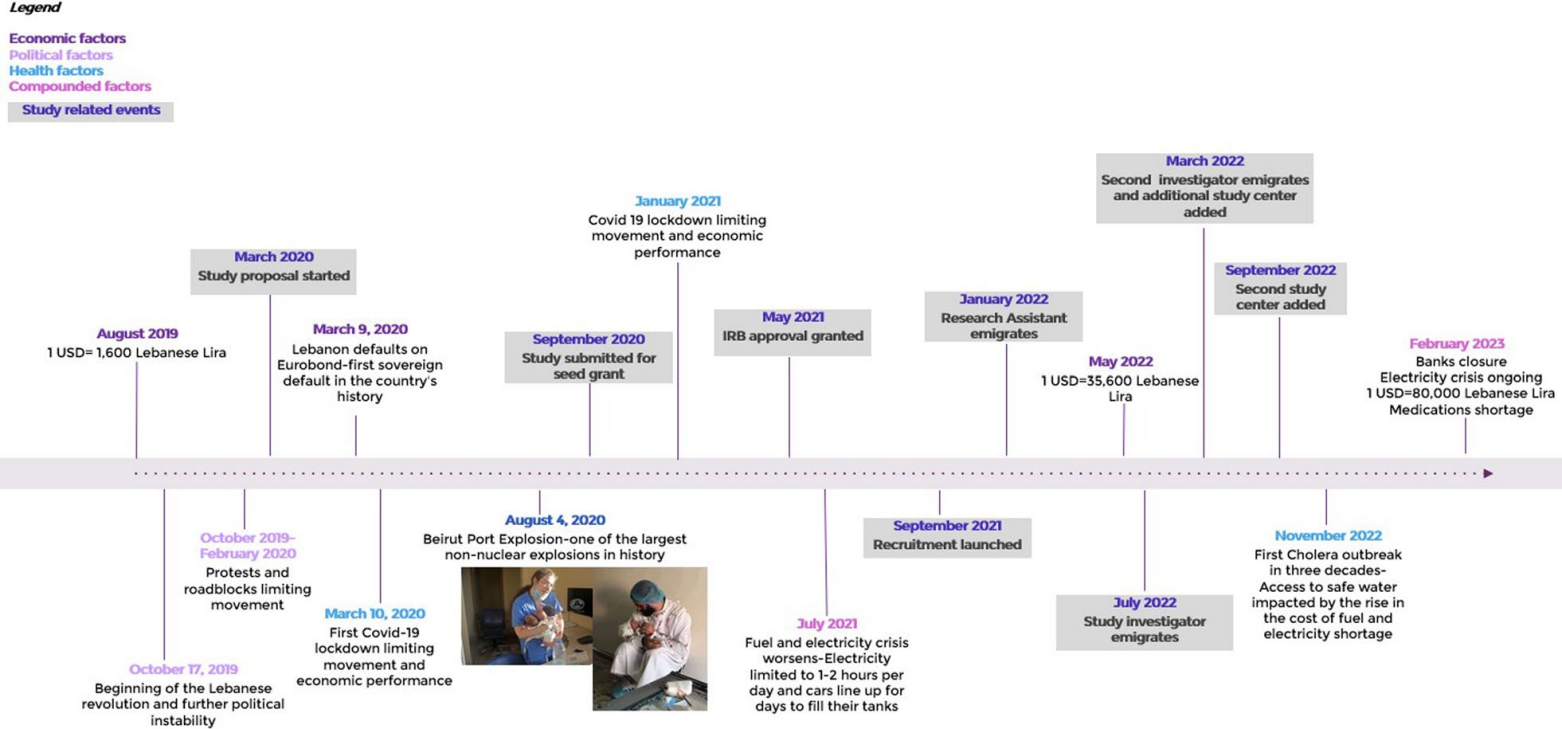
Timeline of a nation’s collapse

Until May 2023, 113 mother-baby pairs have been recruited including 79 full term babies and 34 babies born preterm. Out of those, 59 mother-baby pairs completed the first follow-up visit at 4–6 months, and 26 mother-baby pairs completed the second follow-up visit at 9–12 months (Fig. 2).

#### Sample characteristics

In terms of sociodemographic characteristics at baseline, although 89% of mothers have a university degree, 50% of them were unemployed and 17% of them either did not have insurance or were insured by the national security fund which is a governmental fund that does not cover medical costs in most instances.

When looking at mothers’ health status, more than a third (38%) had complications during their pregnancy, and 24% had other comorbidities. Whereas 48% gave birth via normal vaginal delivery, 26% had an urgent C-section. As expected, mothers who gave birth to a preterm baby had a higher rate of urgent C-sections as compared to mothers of full-term babies. Finally, only 42% of the babies were exclusively breastfed within 24–48 h post birth. Refer to Table 2 for sample characteristics.

#### Economic impact

The effects of the economic crisis on the decreased purchasing power were also evident in our sample. At baseline, almost 6% of mothers of pre-term babies were unable to acquire clothing nor shelter, 3% were unable to acquire sanitation, and almost 9% were unable to acquire education. In terms of healthcare, 1.3% of mothers of full-term babies reported not being able to acquire healthcare as compared to almost 6% of pre-term mothers. The financial instability also affected our ability to collect the needed data. In fact, with the severe fluctuations of the Lebanese Lira rate, several mothers were unable to calculate their average monthly income and 40% were unable to report their income as its value would differ from one day to the other and hence, they were unable to accurately calculate it.

#### Stress and social support

At baseline, 57% of mothers of full-term babies reported moderate to severe stress levels on the PSS10 scale. Whilst mothers of pre-term babies had higher stress levels with 76.4% of them reporting moderate to severe stress levels.

Despite the relatively high levels of stress that were reported, 85% of mothers indicated that they benefit from high perceived social support, and 15% benefit from moderate social support at baseline.

#### Quality of life

In terms of quality of life measured by the WHO-QoL questionnaire (Table 3), different scores were noted between the full term and the preterm group at baseline. Compared to mothers of full-term infants, mothers of preterm infants had lower scores on all the WHOQoL domains: Physical (57.6 vs. 38.2), Psychological (66.3 vs. 55.2), Social (69.2 vs. 59.1), and Environmental (72.7 vs. 69.4).

**Table 3:** Baseline means of continuous variables for 113 Lebanese mother-baby pairs enrolled in the cohort

Means at baseline for continuous variables	Mean (SD)	
	Full term	Pre-term
Mother's age in years	31.6 (4.9)	31.8 (4.9)
Birth weight in grams	2397.3 (1100.3)	1857.5 (695.3)
Apgar at 1 min	8.3 (0.9)	7.9 (1.2)
Apgar at 5 min	9.3 (0.4)	8.9 (0.7)
Parity	0.91 (1.05)	0.86 (1.15)
Number of co-residents	3.2 (1.3)	2.9 (1)
Number of rooms	4.1 (1.4)	3.9 (1.1)
WHOQOL physical	57.6 (18.5)	38.2 (21)
WHOQOL psychological	66.3 (17)	55.2 (15.5)
WHOQOL social	69.2 (19.8)	59.1 (22.4)
WHOQOL environmental	72.7 (17.3)	69.4 (16)
Society scale	7.13 (1.63)	6.50 (2.17)

## Discussion

Establishing a prospective mother and child cohort does not come without its challenges. In a crisis ridden and low resource setting, challenges and barriers are expected to occur more frequently and to have a bigger impact on both the study population and the day-to-day activities of the research project. The establishment of our cohort was done as the crises were unfolding in Lebanon.

In the past few years, the number of Lebanese individuals seeking refuge from the escalating crisis has increased significantly with almost half of the citizens stating that they desire to emigrate [4], with this number increasing to almost two thirds when looking at those between 18 and 29 years old [4].

Another aspect affecting recruitment is the decreased birth rate. From 2017 to 2021, the births rate in Lebanon has decreased by around 24% [25], reaching the lowest rate recorded for the nation. Given the economic situation and political crises, women in Lebanon are becoming more reluctant to get pregnant and are delaying childbearin [1, 25, 33]. Indeed, in one of the study centers, the NICU which used to be fully occupied with 15 beds, is now hosting 3–5 babies at a time at most.

We have also been facing challenges in allocating resources and in identifying healthcare professionals who are able to support with the study. With the exodus of healthcare workers and the lingering effects of the Covid-19 pandemic, healthcare professionals who have remained in Lebanon are overworked and cannot properly dedicate the time to support in research projects. The Lebanese health sector has been badly strained by the effects of the compounding crises. The World Health Organization has estimated in September 2021 that nearly 40% of Lebanon's doctors and almost 30% of nurses had departed since October 2019 [32]. With no expected resolution of the crises any time soon, this number is estimated to have increased as well. Indeed, we have witnessed the effects of this exodus first hand with two of our investigators and one of our research assistants leaving the country over the past 2 years. In developing countries, operational barriers including difficulty in patient recruitment and lack of human research capacity is a major barrier to research which has been reported in several other studies [3]. Targeted recruitment efforts involving community-based recruitment, collaboration with patient advocacy groups and non-governmental organizations, aiming at outpatient health centers and clinics are measures that can be implemented in such a setting to boost recruitment efforts.

Financial challenges were a main barrier faced more and more frequently as the study progressed. Some mothers have shared that the increasingly high costs of consultations, which are being charged in USD rather than the collapsing Lebanese Lira, is leading them to schedule their children’s follow-up visits with paediatricians at tertiary care centers rather than with the paediatricians at private medical centers. The economic collapse along with the COVID 19 pandemic has made healthcare inaccessible to many residents in Lebanon [41]. In a country in which the local currency is in free fall and having lost more than 97% of its value already [35], it has been very challenging to document and report individual financial resources. In such instances, we would recommend resorting to collecting data on proxy measures of financial capabilities, such as assets own, ability to meet needs (such as shelter, food, clothing, education…etc.), and household crowding index.

In addition, limited basic infrastructure such as reliable transportation and stable electricity (as is the case in Lebanon) pose real challenges towards research progress in resource-limited settings. Often, the priorities of research funders do not align with the most urgent needs of low- and middle-income countries [10]. Whilst access to healthcare presents differently in different countries, it is still a universal issue as manifested by the growing demand for sustainable models which separate research costs from other costs [10]. Minimizing the need for transportation for participants by attempting to schedule study related visits alongside healthcare follow-up visit in the same medical center can support in mitigating the impact of limited transportation. If funding is available, providing transportation fees to the participants to and from the visits would also be a helpful measure.

In a context of crisis settings, shedding light on the intricate social determinants and adversities is of utmost importance for guiding resources and interventions. With this backdrop, stress levels are expected to be affected, particularly with mothers of newborns who are undergoing life changing events. Researching in crisis settings is essential for illuminating the complex interplay of social determinants and adversities. With Lebanon at the center of combined upheavals, recruitment and data collection challenges have been substantial.

Our results highlight the need for research funds that prioritize hard to reach populations and on integrating design and measurement approaches that can capture rapid changing social environments and their impact on child outcomes. Limited information on certain socioeconomic indicators is not only a manifestation of the ongoing socioeconomic collapse but it could also be a source of information bias in our sample which is ought to be addressed. We are monitoring participation and attrition rates and documenting reasons for participation refusal and withdrawal. Indeed, collecting information on participation and attrition not only provides insight into potential barriers and how to address them but is also necessary to identify any selection bias. Comparing the characteristics of those who remain enrolled vs those lost to follow up is an important measure to detect selection bias and whether a specific group is particularly impacted as compared to others. In crisis settings, this becomes an even more critical concern, ensuring that resources and interventions can be accurately guided by the research findings.

Our preliminary results showcase significant differences in quality-of-life measures between mothers of preterm babies and those of full-term babies, in a crisis setting. This highlights the intricated social determinants and adversities important for guiding resources & interventions. Mothers are experiencing levels of stress which are increasing with time. Although it is not clear whether this is impacted by the new challenges they are facing as mothers, or the effects of the economic, social, and health crises, or both, nonetheless, the rising challenges and limited availability of resources are expected to lead Lebanon into a mental health epidemic [18].

On average, the mothers in our sample ranked themselves 6.8/10 at the society ranking scale (A scale from 1 to 10 in which mothers subjectively rank their social status as compared to the society they are a part of), keeping in mind that the presented results are of a majority of mothers visiting the top ranked medical center in the capital which attracts communities of higher socioeconomic strata. Our results represent the tip of the iceberg as stronger impact is expected in lower socioeconomic communities. Thus, while building a cohort in a crisis setting, having a representative sample which includes all socioeconomic levels should be given particular attention as these communities are expected to be the most vulnerable and the most affected by the crisis.

Quality research is crucial to address the health needs of those in resource-limited areas, including women and children. However, most research still occurs in high-income countries, and is designed to cater to their specific health priorities. This means that many communities globally lack access to advanced health interventions and lack the needed research specific resources and infrastructure.

### Ethical considerations

Mothers who have just given birth and babies, particularly preterm infants, are particularly vulnerable. In the context of socioeconomic challenges like those faced by Lebanon, conducting research with mothers and babies poses several additional ethical considerations.

All participants gave their written informed consent prior to study inclusion. Due to the vulnerability of the situation, especially in a crisis-hit area, ensuring that consent is freely given without undue pressure or perceived obligation is paramount. The emotional and psychological stress associated with giving birth to a preterm infant might be exacerbated by participating in a study, particularly when inquiring about specific variables that might be associated with developmental outcomes of preterm babies. It is important to reinforce to the mother that she should not feel any guilt or responsibility over socioeconomic factors which are not within her control. Providing equitable access is not only limited to healthcare services. Our research project is giving mothers the chance to assess their infants’ developmental progress, which they would not have otherwise had access to, and should not only target one specific socioeconomic category. In a nation grappling with socioeconomic challenges, we aimed at including multiple medical centers across the country which target different communities and not only those who are easily accessible.

Finally, in the event when a developmental delay is identified through assessment, the child is referred to a specialist. For the mothers whose babies scored low on the Ages & Stages Questionnaire and who needed a referral or to be retested, these were referred to the neonatal and continuity clinic at AUBMC. The clinic offers a comprehensive assessment of infants’ physical and developmental health and involves a team of specialists including a neonatologist, a neonatal fellow, a neonatal nurse, a developmental specialist, and a social worker. Six out of the 25 babies to date who either needed referral or were provided activities and needed to be retested, attended a follow-up visit and 4 of them undertook further developmental assessment with the Bayley scales of infant and toddler development. All these infants were initially recruited at AUBMC and thus were familiar with the health center and its procedures. All the infants recruited at the other centers passed the Ages & Stages Questionnaire. In the event where one of them were not to pass the questionnaire, they would have been referred to the same center. However, in the context of limited resources and limited financial capabilities, some mothers might not possess the ability (financial, logistic, transportation…. etc.) to take their children to specialists, which opens the doors to the ethical dilemma of offering a diagnosis but not the needed support and follow up. Indeed, only 24% of those who needed a follow-up visit did so, despite their familiarity with the healthcare system at the center.

While research in such settings can offer invaluable insights and potential solutions to pressing issues related to vulnerable groups, it is imperative to approach the process whilst respecting ethical considerations and with a commitment to the well-being of the participants and the wider community.

## Conclusion

Establishing a prospective mother and child cohort in a crisis-ridden and low-resource setting like Lebanon presents numerous challenges. The simultaneous occurrence of economic collapse, political instability, decreased birth rates, healthcare worker exodus, financial challenges, and infrastructure limitations has made conducting research in this context exceptionally difficult. There is a pressing need for research funding that prioritizes hard-to-reach populations, that supports building research capacity, and that incorporates adaptable measurement approaches to capture the rapidly changing social environments affecting child developmental outcomes. In Lebanon and similar settings, the challenges faced in establishing and conducting such cohorts serve as a reminder of the imperative to address health disparities in times of crisis and resource scarcity. Upon completion of recruitment and follow up, the study will assess the relationship between social determinants and developmental outcomes. In a country that has been going through one crisis after the other, interventions can be built based on the results and later tested thus guiding resources and management approaches.

## Data Availability

The dataset supporting the conclusions of this article is available in the following repository [https://github.com/rimak18/Building-a-Maternal-and-Child-Cohort-Amidst-Lebanon-s-Socioeconomic-Collapse-Preliminary-Results-/blob/main/Dataset%20for%20manuscript.xlsx].
